# Serologic Evidence of H1 Swine Influenza Virus Infection in Swine Farm Residents and Employees

**DOI:** 10.3201/eid0808.010474

**Published:** 2002-08

**Authors:** Christopher W. Olsen, Lynnette Brammer, Bernard C. Easterday, Nancy Arden, Ermias Belay, Inger Baker, Nancy J. Cox

**Affiliations:** * University of Wisconsin-Madison, Madison, Wisconsin, USA; † Centers for Disease Control and Prevention, Atlanta, Georgia, USA; ‡ College of Medicine, Texas A & M University, College Station, Texas, USA

**Keywords:** swine, influenza, zoonosis, seroprevalence, H1, hemagglutination-inhibition

## Abstract

We evaluated seropositivity to swine and human H1 influenza viruses in 74 swine farm owners, employees, their family members, and veterinarians in rural south-central Wisconsin, compared with 114 urban Milwaukee, Wisconsin, residents. The number of swine farm participants with positive serum hemagglutination-inhibition (HI) antibody titers >40 to swine influenza viruses (17/74) was significantly higher (p<0.001) than the number of seropositive urban control samples (1/114). The geometric mean serum HI antibody titers to swine influenza viruses were also significantly higher (p<0.001) among the farm participants. Swine virus seropositivity was significantly (p<0.05) associated with being a farm owner or a farm family member, living on a farm, or entering the swine barn >4 days/week. Because pigs can play a role in generating genetically novel influenza viruses, swine farmers may represent an important sentinel population to evaluate the emergence of new pandemic influenza viruses.

 Infections with influenza viruses that circulate within the human population are a common and important cause of respiratory disease in people and result in an average of approximately 20,000 deaths and 114,000 hospitalizations per year in the United States alone (1–3). Influenza A viruses also infect animals of a wide variety of other species. In particular, influenza is a common and economically important cause of respiratory disease in pigs (4,5); subclinically infected wild waterfowl provide a global reservoir of influenza A viruses of all 15 hemagglutinin (HA) and 9 neuraminidase (NA) subtypes (6,7).

 The occurrence of H5N1 and H9N2 virus infections among people in Asia in 1997–1999 (8,9) highlighted the potential for avian influenza viruses to cross species barriers to infect humans, but direct avian-to-human transmission of influenza viruses is a rare event. In contrast, the species barrier for transmission of influenza viruses between people and pigs appears to be less stringent, and influenza virus infections in pigs pose important public health concerns at two levels. First, because respiratory tract epithelial cells in pigs contain the sialic acid receptors preferred by both avian (α2,3-N-acetylneuraminic acid-galactose) and human (α2,6-N-acetylneuraminic acid-galactose) influenza viruses (10), pigs are postulated to serve as the “mixing vessel” hosts in which reassortment between avian and human viruses can generate genetically novel viruses with pandemic potential (7,11,12). Reassortment between human and avian influenza viruses produced the 1957 and 1968 pandemic viruses (7). More recently, human-avian reassortant viruses have been isolated from pigs in Europe and, thereafter, from children in the Netherlands (13,14).

 Zoonotic infections of humans with swine influenza viruses, first confirmed by isolation of swine influenza viruses from both pigs and their caretaker on a farm in southern Wisconsin in November 1976 (4), have been diagnosed in the United States, Europe, New Zealand, and Asia (15). However, the total number of zoonotic infections that have been described is relatively small compared to the number of people worldwide involved directly or indirectly in swine farming. Swine farm workers are likely to be routinely exposed to and infected with swine influenza viruses, but only a small percentage of those zoonotic infections are documented. Zoonotic infections may be recognized if information regarding contact with sick pigs is specifically communicated to physicians, if a patient is hospitalized or dies, or if virus isolation is pursued and yields a virus that is antigenically atypical. In most cases, however, swine influenza virus infections in people may not be clinically distinguishable from routine human influenza virus infections. We developed this study to serologically assess the relative level of exposure to classical H1 swine influenza viruses among people involved in swine farming.

## Methods

### Study Population and Design

 Names and contact information for swine farmers living in rural areas of south-central Wisconsin were provided by area swine veterinarians. We contacted these farmers initially by telephone and then, if they were interested in participating, one of the study directors met with them to explain the project’s objectives and procedures. To take part in the study, persons were required to allow home health nurses to collect two blood samples for influenza virus serologic testing and to complete a questionnaire regarding their general health and the nature of their contact with pigs. Participation was also extended to other employees on the farm, spouses and children >7 years of age, and farm veterinarians. A total of 79 participants were initially enrolled, including 76 persons from 22 farms, as well as 3 farm veterinarians. All participants who completed the study were compensated by payment of a $100 honorarium.

 We chose the time period of this study to correspond with the seasonality of swine influenza. In the northern midwestern United States, swine influenza activity is maximal in the late fall and winter (16). Home health nurses visited each participant to administer the study questionnaire, collect an initial preseason blood sample in September 1996, and again to collect a postseason blood sample in April 1997. A total of 114 control serum samples were obtained from a serum bank at the Wisconsin State Laboratory of Hygiene. These samples had been submitted for routine serologic testing from residents of urban Milwaukee, Wisconsin, between August 30, 1996, and March 13, 1997. Because the people from whom control sera were obtained were not specifically enrolled in our study, contacting these persons to gather additional information regarding their health status or activities was neither possible nor ethically appropriate. The use of human participants and control human serum samples in this study was approved by the Human Participant Committees of both the University of Wisconsin-Madison and the Centers for Disease Control and Prevention.

### Questionnaire Topics

 Farm participants were questioned as to their age and sex, their overall health, the nature of their contact with swine, and their influenza virus vaccination history. The specific questions asked of each participant are listed in the questionnaire (Figure).

**Figure:**
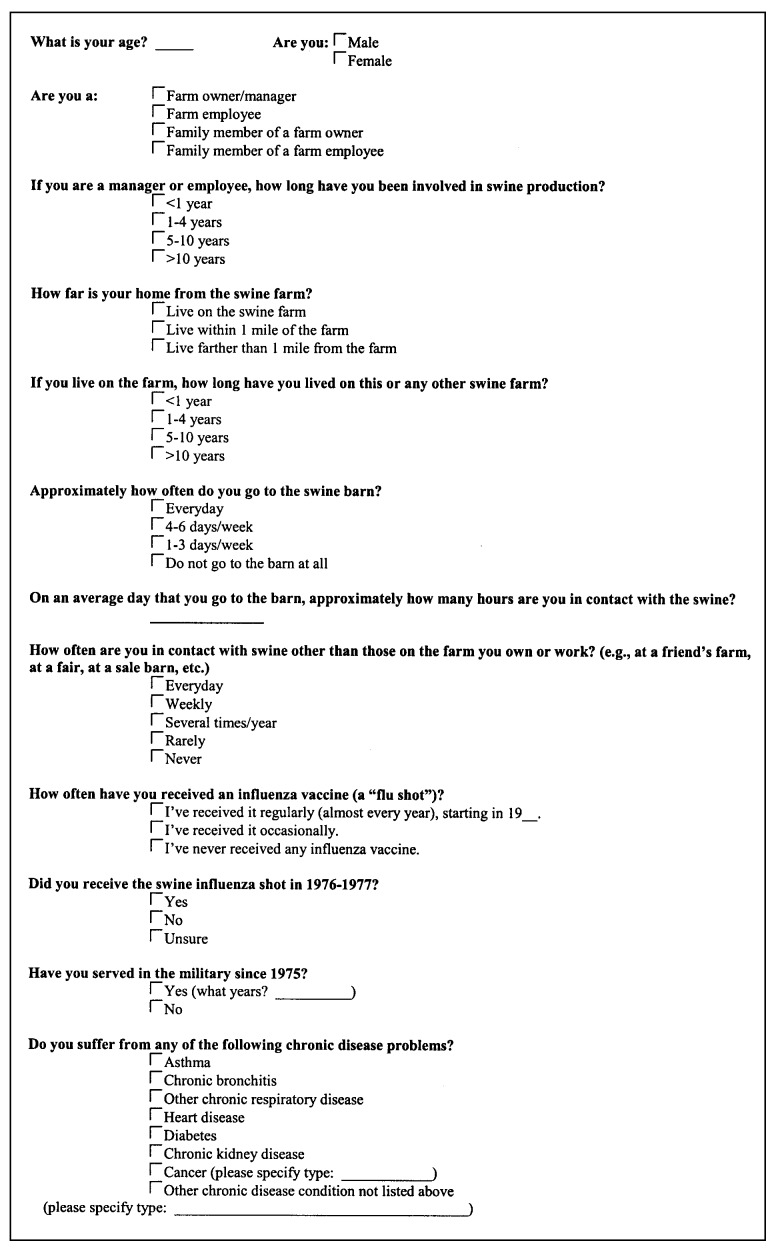
Questionnaire administered to swine farm participants in this study.

### Laboratory Procedures

 Each participant was assigned an ID number so that laboratory samples could be assayed without knowledge of personal identifier information. The human serum samples were treated with receptor-destroying enzyme (Denka Seiken Co., Ltd., Tokyo, Japan) at 37°C for 18 h to eliminate nonspecific inhibitors of hemagglutination, after which the samples were tested for HA-specific antibodies using a standard hemagglutination-inhibition (HI) assay (17). The following strains of human and classical swine influenza A viruses were employed as antigens: A/Johannesburg/82/96 (A/JOH; human H1N1), A/Nanchang/933/95 (A/NAN; human H3N2), A/Nebraska/01/92 (A/NEB; zoonotic human isolate of swine H1N1 influenza virus), and A/Swine/Indiana/1726/88 (Sw/IND; swine H1N1). Control sera included sheep anti-human H1 and anti-human H3 subtype-specific sera, ferret anti-A/NEB, and normal sheep serum. Each human serum sample was also assayed without added viral antigen (serum-only control) in parallel with the virus-specific assays. All HI assays were run simultaneously, and HI titers were defined as the reciprocal of the highest dilution of serum that inhibited virus-induced hemagglutination of a 0.5% solution of chicken red blood cells. To calculate geometric mean titers (GMTs) for individual cohorts, we included values for virus-specific titers only if they were greater than the corresponding serum-only control values. Fourfold rises in titer were examined for the study participants by comparing their pre and postseason serum antibody titers.

### Statistical Analyses

 The numbers of sera with an HI titer >40 to either swine virus were compared among the preseason farm participant samples versus the urban control samples by chi-square analysis. The GMTs of the samples from preseason farm participants were compared to the GMTs of the urban control sera by using Wilcoxon rank sum analysis with normal approximation. We also evaluated associations between preseason seropositivity to swine influenza viruses at HI titers >40 or >80 among the farm participants and specific aspects of swine exposure or other variables. These associations were then examined for statistical significance by chi-square or two-sided Fisher’s exact analyses. Multivariate analysis was not done because of the small numbers of participants with elevated preseason titers to swine influenza viruses (17 participants with titers >40; 11 participants with titers >80).

## Results

 Seventy-nine swine farming participants were initially enrolled, including 20 farm owners (ages 28–59 years), 14 spouses of farm owners (34–57 years), 12 children of farm owners (7–21 years), 21 farm employees (19–43 years), 9 spouses of farm employees (21–39 years), and 3 veterinarians (29–54 years). In total, the farm participants included 44 males of median age 37 (range 13–59 years) and 35 females of median age 34 (range 7–57 years). Of these participants, two people moved out of the area during the study and did not participate further, and three people chose to withdraw from the study before their second blood samples were collected. The preseason samples from these five participants were not included in the analyses. Two participants (both with elevated titers to swine viruses) did not complete the questionnaire. Serologic data from these two participants were included in the comparison of preseason titers of farm participants versus controls but could not be used to assess variables associated with seropositivity to swine viruses. The control sera were from 54 males of median age 32 (range 2–58 years) and 60 females of median age 34 (range 4–54 years).

 To interpret the HI titers as showing differences in exposure to swine versus human influenza viruses, we first had to demonstrate that no minimal serologic cross-reactivity in HI assays between the human and swine reference strains existed. We compared HI titers by using virus-specific sheep and ferret reference sera (Table 1) and clearly showed no serologic cross-reactivity in HI assays between the human H1N1, human H3N2, and swine H1N1 viruses. The HI titers to homologous viruses were 320–640, whereas titers to heterologous viruses (H1 vs. H3 or human H1 vs. swine H1) were only 5–10.

**Table 1:** Hemagglutination-inhibition titers of control sera to reference virus strains used in this study

	Reference influenza A viruses			
Control serum	A/Johannesburg/82/96 (A/JOH) (human H1N1 virus)	A/Nanchang/933/95 (A/NAN) (human H3N2 virus)	A/Nebraska/01/92 (A/NEB) (zoonotic swine H1N1 virus)	A/Swine/Indiana/1726/88 (Sw/IND) (swine H1N1 virus)
Sheep anti-human H1N1^a^	640	10	5	5
Sheep anti-human H3N2^b^	5	320	5	5
Ferret anti-A/NEB	5	10	640	320
Ferret anti-swine H1N1^c^	5	10	320	640
Normal sheep serum	10	10	5	5

^a^Produced by immunization of sheep with A/Taiwan/1/86 and A/Texas/36/91 (H1N1). ^b^Produced by immunization of sheep with A/Shangdong/9/93, A/Johannesburg/33/94, and A/Nanchang/933/95 (H3N2). ^c^Produced by immunization of ferrets against A/Swine/Wisconsin/01/88 (H1N1).

 The preseason serum samples from 17/74 farm participants had HI titers >40 (titer range 40–160) against either A/NEB or Sw/IND; 15/17 were seropositive to both swine viruses. These participants included seven farm owners (range 41–55 years), seven family members of farm owners (range 7–54 years), a 33-year-old farm employee, a 38-year-old family member of a farm employee, and a 43-year-old veterinarian. In contrast, only 1/114 of the urban control serum samples (from a 41-year-old) had a positive HI titer against a swine virus (HI titer=40 against only A/NEB). The difference in the number of seropositive samples between the farm participant and urban control cohorts was statistically significant (p<0.001). Similarly, the GMTs of the preseason serum samples from the farm participants to both swine-lineage viruses (A/NEB and Sw/IND) were significantly higher (p<0.001) than the GMTs of the samples from the urban control participants (13.2 vs. 5.1 and 15.7 vs. 5.4, respectively). In contrast, the farm participants’ GMTs to the reference human H1 (A/JOH) and H3 (A/NAN) viruses were not significantly different from those of the urban control samples (Table 2).

**Table 2:** Geometric mean titers of preseason serum samples from farm participants and urban control serum samples

	Reference influenza A viruses			
Participants	A/Johannesburg/82/96 (A/JOH) (human H1N1 virus)	A/Nanchang/933/95 (A/NAN) (human H3N2 virus)	A/Nebraska/01/92 (A/NEB) (zoonotic swine H1N1 virus)	A/Swine/Indiana/1726/88 (Sw/IND) (swine H1N1 virus)
Farm participants	15.3	8.6	13.2^a^	15.7^a^
Urban control participants	14.2	8.0	5.1	5.4

^a^p>0.0001 (Wilcoxon rank sum analysis with normal approximation).

 Only three farm participants demonstrated fourfold rises in titer to either of the swine viruses. These rises were not associated with illness in either the human participants or the pigs on their farms.

 Each of the variables on the questionnaire (Figure) was investigated for association with preseason sample seropositivity to Sw/IND at HI titers >40 or >80, A/NEB at HI titers >40 or >80, and either swine virus at HI titers >40 or >80. The variables associated with seropositivity to either swine virus at HI titers >40 or >80 and the statistical strength of those associations are shown in Table 3. (Results for seropositivity to each individual swine virus are not shown but were consistent with the summary statistics presented in Table 3.) Being a farm owner, being part of a farm family (a farm owner or a farm owner’s family member), living on a swine farm, and going into a swine barn >4 days/week were all associated with seropositivity to swine influenza viruses. Beyond these factors of pig contact, being >50 years of age (but not >36 years of age, the median age of the farm participants in the study) was associated with swine virus seropositivity; having received the swine flu vaccine in 1976–77 or having ever received any influenza virus vaccine was also associated with swine virus seropositivity. (All four persons who received the swine influenza vaccine also reported having received other influenza virus vaccines.)

**Table 3:** Variables associated with seropositivity to swine influenza viruses among the farm participants and the statistical strength of these associations ^a^

Variable	HI titer >40^b^	HI titer >80^b^
Being a farm owner	p=0.04	p=0.02
Being a farm owner or the family member of a farm owner	p=0.03	p=0.02
Living on a swine farm	(p = 0.07)	p=0.04
Going into a swine barn >4 days/week	(p = 0.12)	p=0.04
Age >50 yrs	p=0.02	p=0.03
Having received the swine flu vaccine in 1976–77	p=0.02	(p = 0.44)
Ever having received any influenza virus vaccine	p=0.03	(p = 0.19)

^a^P values determined by chi-square or two-sided Fisher’s exact analyses; p values >0.05 cut-off for significance are shown in parentheses. ^b^To either swine virus. Abbreviation used: HI, hemagglutination-inhibition.

## Discussion

 Although zoonotic infections with swine influenza viruses have been documented previously (4,15), the results of the present study strongly support the hypothesis that people associated with swine production are infected with swine influenza viruses more regularly than the small number of zoonotic infections in the literature would suggest. Previous studies by Kluska et al. (18), Woods et al. (19), and Schnurrenberger et al. (20) in the 1960s suggested increased rates of infection among persons in contact with pigs or working with swine influenza viruses. In this study, we specifically associated factors related to a person’s degree of contact with pigs to seropositivity to swine viruses. The number of hours per day spent in the barn was not a factor of significance, suggesting that the overall frequency of pig contact is a more important consideration than the length of contact at any one time. This lack of significance is consistent with the fact that influenza virus infections in pigs occur sporadically, and pigs generally only shed virus for approximately 7 days after infection (21). During the course of this study, pigs on only one farm were reported to exhibit signs of influenza-like illness. Influenza viruses were not isolated from nasal swab samples collected from these pigs.

 Two factors not directly related to swine contact were also statistically associated with seropositivity to swine viruses in our study. First, being >50 years of age was associated with swine virus seropositivity. In an earlier study, Schnurrenberger et al. (20) collected samples in 1966 from abattoir workers, pork producers, swine exhibitors at a state fair, and veterinarians; they also found an association between age and seropositivity to a classical H1N1 swine influenza virus. In that study, the major impact of age was apparent for people born before 1920, suggesting an effect from exposure to the swine-like 1918 pandemic influenza virus (22–24). We could not fully separate the effects of age and exposure over time to swine. All of the participants >50 years of age were farm owners or farm family members. However, several factors indicate that, although age may play a role in seropositivity to swine viruses, exposure to swine is a more dominant factor. Farmers and their family members were significantly more likely than employees and their family members to have elevated titers to swine viruses; farmers and their families were also more likely to have exposure to swine and to be exposed over a longer period of time. Specifically, 88% of the farm owners and their families lived on the swine farm, compared to 7% of the employees and their families. Furthermore, of the farm owners and their families who lived on the farm, 77% had lived there >11 years. Farm owners had significantly more years in swine production than their employees (Mantel-Haenszel chi-square test for trend, p<0.001). Among younger study participants (<50 years of age), 21% and 18% of the farm family members had titers of >40 and >80 to swine viruses, respectively, compared to only 7% and 4% for employees and their family members. Although these differences among the younger participants were not statistically significant (p=0.09 to 0.14), they are consistent with the pattern of elevated titers seen among those with a higher level of exposure to pigs. Finally, our control population was of the same overall age distribution as our farm participants, yet only a single 41-year-old person among these controls was seropositive to a swine virus.

 A second factor unrelated to swine contact significantly associated with swine virus seropositivity was having received either the swine influenza virus human vaccine in 1976–77 or ever having received any human influenza virus vaccine. However, vaccination status alone most likely did not determine seropositivity to swine viruses among our farm participants. Vaccination was only associated with seropositivity at a titer >40, but not at >80. Although we do not have historical data for our urban control samples, we have no a priori reason to suspect that these people would have had substantially different vaccination histories. However, only 1/114 of these participants demonstrated a titer >40 to a swine virus. Likewise, the proportions of employees and their family members who received the swine influenza vaccine (4%) or other influenza vaccines (32%) were not significantly different from the proportions of farm family members who received the swine influenza vaccine (6%) or other influenza vaccine (22%), but farm family members were significantly more likely to have elevated titers to swine viruses. Regarding having received the 1976–77 swine influenza vaccine, antibodies produced against that vaccine would not likely be present at detectable levels 20 years later. However, studies have shown that vaccination with more recent human influenza A (H1N1) viruses can boost titers to swine-like viruses in those previously exposed (25). Therefore, the statistical association between seropositivity to swine viruses and vaccination likely reflects a vaccine-induced boosting of antibody titers in persons previously exposed to a swine influenza virus. Because of the overall low numbers of participants with elevated titers to swine viruses, we were not able to perform meaningful multivariate analysis of the data to definitively segregate the effect of vaccination history (or age) from other variables.

 Because a relatively small number of zoonotic swine influenza virus infections have been documented by virus isolation, whether infections with swine influenza viruses are clinically different than infections with routine human influenza viruses remains unclear. Our data suggest that aggressively pursuing virus isolation when people involved in swine farming have influenza-like illnesses would be valuable. In this way, retrospective studies of the clinical appearance of a larger number of zoonotic swine influenza cases may be possible.

 Our findings suggest a second issue. Pigs may serve as hosts for the adaptation of avian viruses to replication in mammalian species (26). In addition, pigs are clearly recognized as hosts in which genetic reassortment between human and avian viruses can produce novel strains of pandemic potential (7,11,12). While this concern has historically been thought to be most important in the “Asia epicenter” (12,27), avian H1N1 viruses have spread widely within the swine population of northern Europe since 1979 (7,28–31), avian H4N6 viruses were isolated from pigs in Canada in 1999 (32), and human/swine/avian reassortant H3N2 (33–35) and H1N2 (36,37) viruses have spread widely within the swine population of the United States since 1998. Given the apparent frequency with which swine farm workers in our study were exposed to influenza viruses from pigs, more closely monitoring such persons as potential sentinels for the emergence of novel influenza viruses from the swine populations of developed countries with extensive swine-raising industries may be prudent.
